# Expression patterns for nicotinic acetylcholine receptor subunit genes in smoking-related lung cancers

**DOI:** 10.18632/oncotarget.18948

**Published:** 2017-07-04

**Authors:** Anna Bordas, José Luis Cedillo, Francisco Arnalich, Isabel Esteban-Rodriguez, Laura Guerra-Pastrián, Javier de Castro, Carolina Martín-Sánchez, Gema Atienza, Carmen Fernández-Capitan, Juan José Rios, Carmen Montiel

**Affiliations:** ^1^ Departamento de Farmacología y Terapéutica, Facultad de Medicina, Universidad Autónoma de Madrid, Spain; ^2^ Servicios de Medicina Interna, Hospital Universitario La Paz, Madrid, Spain; ^3^ Anatomía Patológica, Hospital Universitario La Paz, Madrid, Spain; ^4^ Oncología Médica, Hospital Universitario La Paz, Madrid, Spain; ^5^ Instituto de Investigaciones Sanitarias IdiPAZ, Madrid, Spain

**Keywords:** nAChRs, NSCLC, squamous cell carcinoma of the lung, lung adenocarcinoma, tobacco

## Abstract

Cigarette smoking is associated with increased risk for all histologic types of lung cancer, but why the strength of this association is stronger for squamous cell carcinoma than adenocarcinoma of the lung (SQC-L, ADC-L) is not fully understood. Because nicotine and tobacco-specific nitrosamines contribute to carcinogenesis by activating nicotinic acetylcholine receptors (nAChRs) on lung tumors and epithelial cells, we investigated whether differential expression of nAChR subtypes in these tumors could explain their different association with smoking. Expression of nAChR subunit genes in paired tumor and non-tumor lung specimens from 40 SQC-L and 38 ADC-L patients was analyzed by quantitative PCR. Compared to normal lung, both tumors share: i) transcriptional dysregulation of *CHRNA3/CHRNA5/CHRNB4* (α3, α5, β4 subunits) at the chromosomal locus that predisposes to lung cancer; and ii) decreased expression of *CHRFAM7A* (dupα7 subunit); this last subunit negatively modulates α7-nAChR activity in oocytes. In contrast, *CHRNA7* (α7 subunit) expression was increased in SQC-L, particularly in smokers and non-survivors, while *CHRNA4* (α4 subunit) expression was decreased in ADC-L. Thus, over-representation of cancer-stimulating α7-nAChR in SQC-L, also potentiated by smoking, and under-representation of cancer-inhibiting α4β2-nAChR in ADC-L could explain the different tobacco influences on the tumorigenic process in each cancer type.

## INTRODUCTION

Lung cancer is the leading cause of cancer-related deaths worldwide [1]. There are two major categories of lung cancer, non-small cell lung carcinoma (NSCLC) and small-cell lung carcinoma (SCLC), respectively accounting for 75–85% and 15–25% of all lung cancer cases. Lung adenocarcinoma (ADC-L) and squamous cell carcinoma of the lung (SQC-L) are the two major histologic types of NSCLC, which also includes large-cell lung carcinoma (LCLC) as a minor type.

Numerous epidemiologic and laboratory-based studies have confirmed that cigarette smoking is associated with increased risk for all major histological types of lung cancer, which is understandable given that tobacco contains more than 70 known carcinogens [2]. However, it is surprising how much the strength of the above association varies between the different histological tumor types, being stronger with SCLC and SQC-L than LCLC and ADC-L [3]. One of the hypotheses to justify the variation in the risk associated with smoking for one or the other tumor type, is related to the location and more-or-less intimate exposure of the tumor to inhaled tobacco smoke and its carcinogenic components. This hypothesis would explain why the more peripheral tumors (ADC-L and LCLC) show weaker associations with smoking than the more central tumors (SQC-L or SCLC) [4]. However, the above proposal does not exclude a second, so far never-explored possibility that would imply tumor-dependent differential expression of target receptors for the carcinogenic components of tobacco.

Tobacco smoke contains multiple classes of carcinogens, including polycyclic aromatic hydrocarbons and the nicotine-derived nitrosamines 4-(methylnitrosamino)-1-(3-pyrydyl)-1-butanone (NNK) and N-nitrosonornicotine (NNN). By inducing the formation of DNA adducts and causing mutations in vital cancer suppressor genes (i.e. *Rb, p53* and *K-Ras*), these carcinogens eventually initiate carcinogenesis [5]. At the same time, the addictive components of tobacco, nicotine and its carcinogenic derivatives NNK and NNN, also significantly contribute to the oncogenic process after binding and activating cell surface nicotinic acetylcholine receptors (nAChRs) expressed in neuronal and non-neuronal cells, including the cancer cells themselves [6]. Stimulating the receptors in the cancer cells leads to downstream activation of multiple signaling cascades that promote cancer cell survival, proliferation, angiogenesis, migration and metastasis in a tumor-specific manner [7–10]. The nAChRs are complex structures composed of five transmembrane subunits arranged around a central ion pore [11]. To date, several alpha (α3-α7, α9) and beta (β2 and β4) nAChR subunits have been identified in primary lung tumors and human lung cancer cell lines [see Ref. 12 and references therein]. Accordingly, it is likely that the above tumors express homomeric α7- and α9-nAChRs (composed of five identical α7 or α9 subunits) and heteromeric α3β4α5-, α3β2α5- and α4β2-nAChRs (constituted by different combinations of α and β subunits).

The different subunit compositions of nAChR determine its functional and pharmacological characteristics as well as its pathophysiological role. In relation to this last aspect, α9-nAChRs are known to play an important role in human breast cancer progression [13], while α7-nAChR is the main subtype responsible for the nicotine-mediated proliferative, pro-angiogenic and pro-metastatic effects in human NSCLC [14–17]. In contrast to the α7-nAChR-mediated stimulatory effects on cancer growth, heteromeric α4β2-nAChR seems to be one of the major inhibitors of cancer development and progression through its release of GABA, which blocks the cancer-stimulating effects of β-adrenergic receptors by inhibiting cAMP [18]. It is interesting to note that chronic exposure to nicotine, and its carcinogenic derivative NNK, increases cancer-stimulating α7-nAChR expression and desensitizes cancer-inhibiting α4β2-nAChR in cancer cells in the lungs and pancreas [16, 19, 20]. This means that smoking activates cancer-stimulating nAChRs in the cells.

Genes *CHRNA3*, *CHRNA4*, *CHRNA5*, *CHRNA6*, *CHRNA7*, *CHRNA9*, *CHRNB2* and *CHRNB4*, located across different chromosomes, respectively encode for the α3, α4, α5, α6, α7, α9, β2 and β4 nAChR subunits expressed in lung cancer cells [see Ref. 12 and references therein]. Several genome-wide association studies (GWAS) have identified a susceptibility locus for human lung cancer at chromosome 15q24-25, which contains three of the above genes: *CHRNA3*/*CHRNA5*/*CHRNB4* [21–23]. The discovery of a new hybrid gene *(CHRFAM7A)* in the human genome, but not in that of other higher primates, is also noteworthy. The hybrid gene results from the fusion of a partial duplication of the *CHRNA7* gene with the *FAM7A* gene [24, 25]. Moreover, the *CHRFAM7A* transcript, named dupα7 mRNA, has been identified in brain (hippocampus, cortex, corpus callosum, thalamus, putamen, caudate nucleus and cerebellum) and in the periphery (peripheral blood mononuclear cells, lymphocytes and synoviocytes) [see Ref. 26 and references therein]. Moreover, our group performed the first study on the functional role of the new dupα7-nAChR subunit heterologous expressed in *Xenopus* oocytes, demonstrating that it behaves as a negative endogenous regulator of α7-nAChR activity [26]. Given the prominent role of α7-nAChR in the development of lung cancer, it is feasible that dupα7 could act as an endogenous suppressor of tumor growth mediated by α7-nAChRs, as long as this atypical subunit was expressed in the tumor. To date, there have been no data in the literature on this question.

The present study aims to determine whether differences in the expression pattern for the genes encoding nAChR subunits that make up target receptors for the carcinogenic components of tobacco could explain the variability of the influence of tobacco in the two major histologic types of NSCLC. To address this issue, we have used real-time quantitative polymerase chain reaction (qPCR) to examine normalized or absolute mRNA expression levels of several nAChR subunits in lung biopsies from 40 patients with SQC-L and 38 patients with ADC-L. Each patient provided matched specimens, one of non-tumor lung tissue and another from their lung tumor.

## RESULTS

### Demographic and clinical characteristics of patients

Seventy-eight NSCLC patients were included in the study and their demographic and clinical characteristics are summarized in Table 1. Patients were grouped according to their histologic tumor type [40 SQC-L (51.3%) and 38 ADC-L (48.7%)], gender (with a majority of men in both tumor types) and age (87% of SQC-L and 92.1% of ADC-L patients were over 60 years old). Most SQC-L patients were smokers (87.5%) at the time of diagnosis with a history of tobacco consumption averaging at least 20 packs per year, while more than half of the ADC-L patients (55.3%) were non-smokers or second-hand smokers. The tumor differentiation grade according to the 2015 WHO classification of lung cancer [27] and long-term outcomes are also summarized in Table 1.

**Table 1:** Demographic and clinical characteristics of NSCLC patients

Histologic type	SQC-L^a^	ADC-L^a^
**Number of tumor biopsies**	40 (51.3%)	38 (48.7%)
**Gender**		
Male	32 (80%)	25 (65.8%)
Female	8 (20%)	13 (34.2%)
**Age**		
< 60	5 (12.5%)	3 (7.9%)
> 60	35 (87.5%)	35 (92.1%)
**Tobacco habit**		
Never smoker	3 (7.5%)	11 (29.0%)
Second-hand smoker	2 (5.0%)	10 (26.3%)
Smoker	35 (87.5%)	17 (44.7%)
**Differentiation grade**		
Well/Moderate	22 (55%)	18 (47.4%)
Poor	18 (45%)	20 (52.6%)
**Clinical outcome**		
Disease-free survival (median)	47 months	54 months
Five-year survival rate (%)	60.0	68.4

^a^A non-tumor lung biopsy resected from each patient is analyzed in parallel to the paired tumor biopsy.

### Expression of nAChR subunit gene in tumor biopsies of SQC-L and ADC-L patients

**Figure 1:**
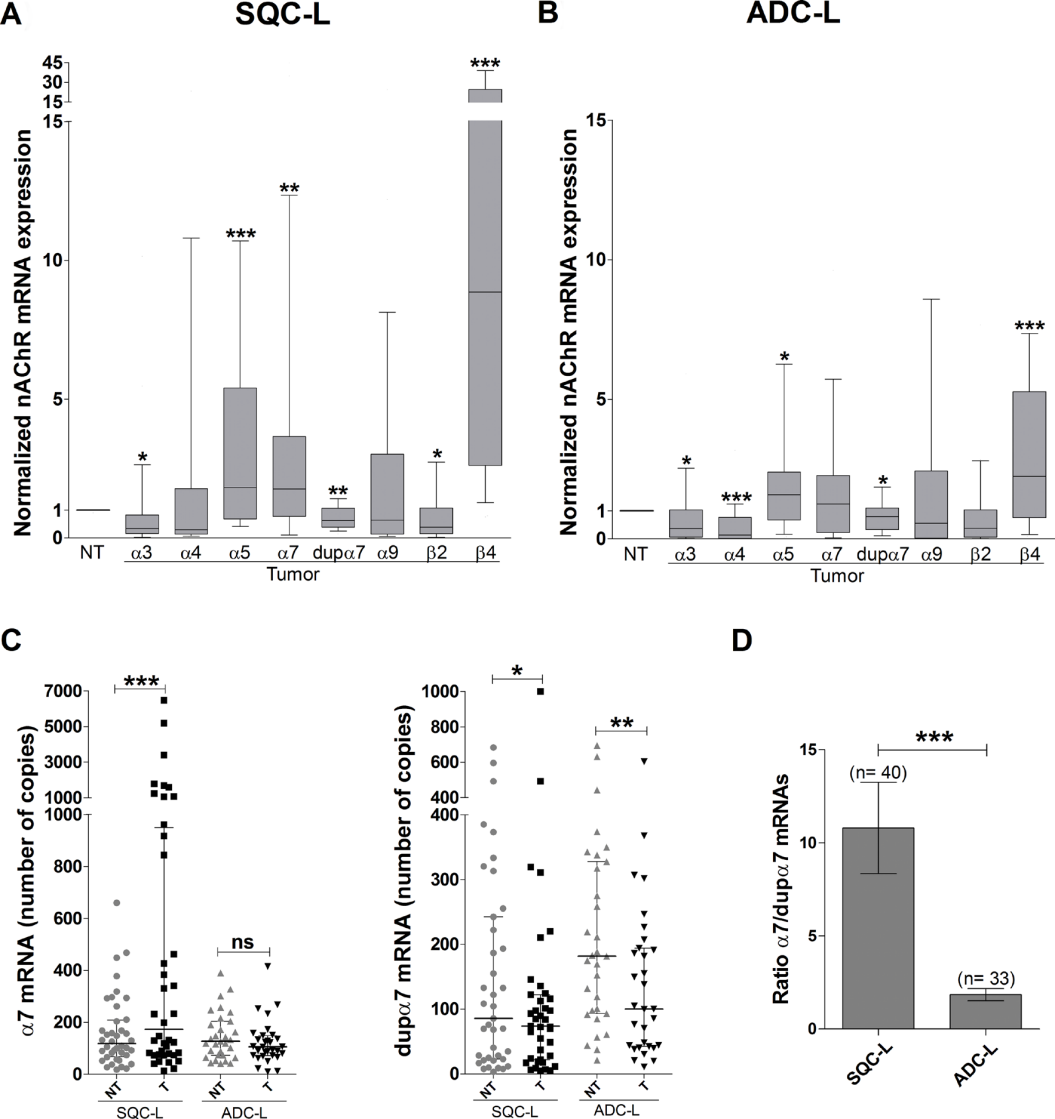
Expression analysis of different nAChR subunit genes in lung tumor biopsies from NSCLC patients Expression of each gene was determined by quantitative PCR in 40 patients with SCQ-L (**A**) and 38 patients with ADC-L (**B**). Each value, obtained in triplicate and representing an average of three separate determinations, was normalized with respect to the expression of the same gene in the corresponding paired non-tumoral (NT) sample from the same patient (referred to as 1). Data are represented as a box-and-whisker plot; the line within each box shows the median expression of each subunit, upper and lower edges of the box represent the 75th and 25th percentile, and the ends of the whiskers the 90th and 10th percentile, respectively. The Wilcoxon test for paired samples was used for data analysis. *p < 0.05, **p < 0.01 and ***p < 0.001 compared to the paired non-tumor sample. (**C**) Dot plots representing the absolute expression number of mRNA copies for the α7 and dupα7 expressed in paired non-tumor (NT) and tumor (T) lung biopsies of 40 patients with SQC-L and 33 patients with ADC-L; panels show the median and interquartile ranges for each gene analyzed. (**D**) The α7/dupα7 ratio was calculated on the basis of the number of copies of both transcripts expressed in the tumor biopsies of the patients. Wilcoxon (C) and Mann-Whitney (D) tests were used for data analysis. *p < 0.05; **p < 0.01; ***p < 0.001, ns = not significant.

Gene expression of nAChR subunits was analyzed in matched tumor and non-tumor lung specimens from each patient included in the study. The upper panels of Figure 1A and 1B show the gene expression for each subunit in the tumor sample normalized with respect to its non-tumor partner for both groups of patients. The results for SQC-L tumors showed a significant increase in α5, α7 and β4, together with a significant decrease in α3, dupα7 and β2 mRNA levels (Figure 1A). We also found that ADC-L and SQC-L tumors shared significant changes in the expression of some nAChR genes (increased α5 and β4 and reduced α3 and dupα7 mRNA levels). However, differently from the latter, ADC-L tumors showed a significant reduction in α4 mRNA level without significant changes in the expression of α7 or β2 mRNAs (Figure 1B).

Our data concerning the normalized α7 and dupα7 mRNA expression in tumors of SQC-L and ADC-L patients were subsequently corroborated by the absolute expression values (number of mRNA copies) of both messengers determined in paired tumor and non-tumor biopsies from the patients (Figure 1C). The results revealed that, compared to non-tumor specimens, only SQC-L tumors showed a significant increase in absolute α7 mRNA expression, but that both tumor types shared a significant reduction in the number of dupα7 mRNA copies. Using the absolute expression values of both messengers in the SQC-L and ADC-L tumors, we calculated the quotient of α7 *versus* dupα7 mRNA molecules in each histological type as a α7/dupα7 ratio. The results demonstratethat, in comparison to ADC-L, SQC-L tumors contain a significantly higher proportion of α7 than dupα7 mRNA molecules (Figure 1D).

### Frequency distribution and correlation analysis of α7 expression *versus* expression of each of the remaining nAChR subunits in SQC-L and ADC-L tumors

**Figure 2:**
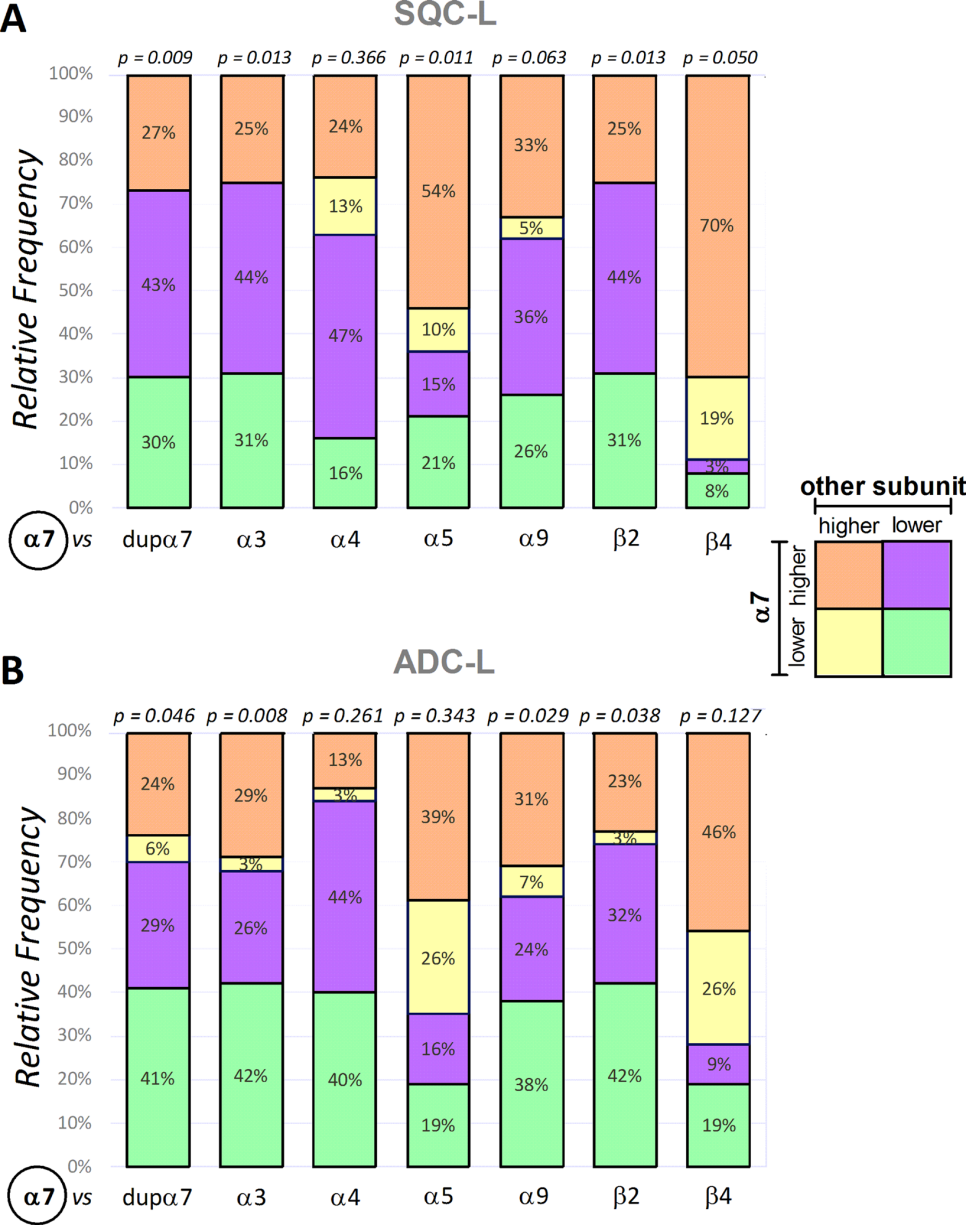
Frequency distribution of α7 expression *versus* expression of each of the remaining nAChR subunits in the SQC-L and ADC-L tumors The stacked bar charts represent the frequency distribution, in SQC-L or ADC-L tumors, for several pairs of categorical variables (α7 *versus* another nAChR subunit), each with two categories (higher or lower mRNA expression in the tumor compared to its paired non-tumor sample). Insert shows the categories identified with different colors. The numbers inside the stacked bars represent the percentage of patients in each segment. Fisher's exact test was used for analysis; statistical significance is shown at the top of the bar.

To obtain complementary information for that provided by Figure 1 in the groups of SQC-L and ADC-L patients, we next analyzed the frequency distribution for two categorical variables (α7 *versus* another nAChR subunit), each with two categories (higher or lower mRNA expression in the tumor compared to its paired non-tumor sample) in each group. The α7 subunit was chosen as the fixed variable for comparison against each of the remaining variables (another nAChR subunit) because the α7-nAChR subtype is the most directly involved in tobacco-induced tumorigenesis. With the qualitative data from above, we produced a contingency table for each tumor type that was later transformed into the corresponding “stacked bar chart” (Figure 2) to facilitate visual comparison between categories (see the insert with the colored categories to the right of the figure). The numbers inside the stacked bars correspond to the percentage of patients in each segment.

The results in SQC-L patients show a statistically significant relationship between α7 mRNA expression and expression of dupα7, α3, α5, β2 or β4 mRNA (panel A). In addition, we found that the most frequent distribution in these patients was the one with increased expression of α7 mRNA in their tumors, which was accompanied by a decreased expression of dupα7 (43% of the patients, purple), α3 (44%, purple), β2 (44%, purple), or increased expression of α5 (54%, orange) and β4 (70%, orange). It should be noted that tumor levels of dupα7, α3 and β2 mRNAs are decreased and those of α5 and β4 increased in most SQC-L patients (73%, 75%, 75%, 64% and 89%, respectively).

Figure 2B shows that for patients with ADC-L, the frequency distribution for each pair of variables differs from that found in SQC-L patients. Therefore, the most common distribution in ADC-L patients corresponds to that with diminished expression of α7 and the remaining related variables [dupα7 (41%), α3 (42%), α9 (38%) and β2 (42%) = green], in their tumors. It is also noteworthy that, regardless of their α7 expression, most ADC-L patients showed reduced tumor expression of dupα7, α3, α9 and β2 (70%, 68%, 62% and 74%, respectively). The statistical analysis of our data does not detect an association between α7 and α4 expression in ADC-L tumors. However, and curiously, most ADC-L patients (84%) present a down-regulation of α4 subunit gene expression that is accompanied by either up-regulation (in 44%) or down-regulation (in 40%) of α7 expression.

Our results also reveal the significant correlation between α7 mRNA expression levels and the level of expression of other nAChR subunits (dupα7, α3 and β2) in both tumor subtypes (Table 2). Moreover, α7 expression levels also correlate with those of α5 mRNA in SQC-L, and with those of α4, α9 and β4 mRNAs in ADC-L tumors.

**Table 2:** Correlation between expression levels of α7 and other nAChR subunits in tumors from NSCLC patients

	dupα7	α3	α4	α5	α9	β2	β4
**SQC-L → α7**	0.395 *(p = 0.012)*	0.537 *(p = 0.0001)*	−0.221 *(ns)*	0.506 *(p = 0.001)*	0.302 *(ns)*	0.438 *(p = 0.005)*	0.281 *(ns)*
**ADC-L → α7**	0.548 *(p = 0.001)*	0.676 *(p = 0.0001)*	0.475 *(p = 0.008)*	0.246 *(ns)*	0.636 *(p = 0.0001)*	0.685 *(p = 0.0001)*	0.387 *(p = 0.031)*

Values represent the Spearman correlation coefficients. The statistical significance is shown in parentheses, ns = not significant.

### Association of smoking, tumor differentiation grade, and gender with the expression levels of several nAChR subunit genes in tumor biopsies from SQC-L and ADC-L patients

**Figure 3:**
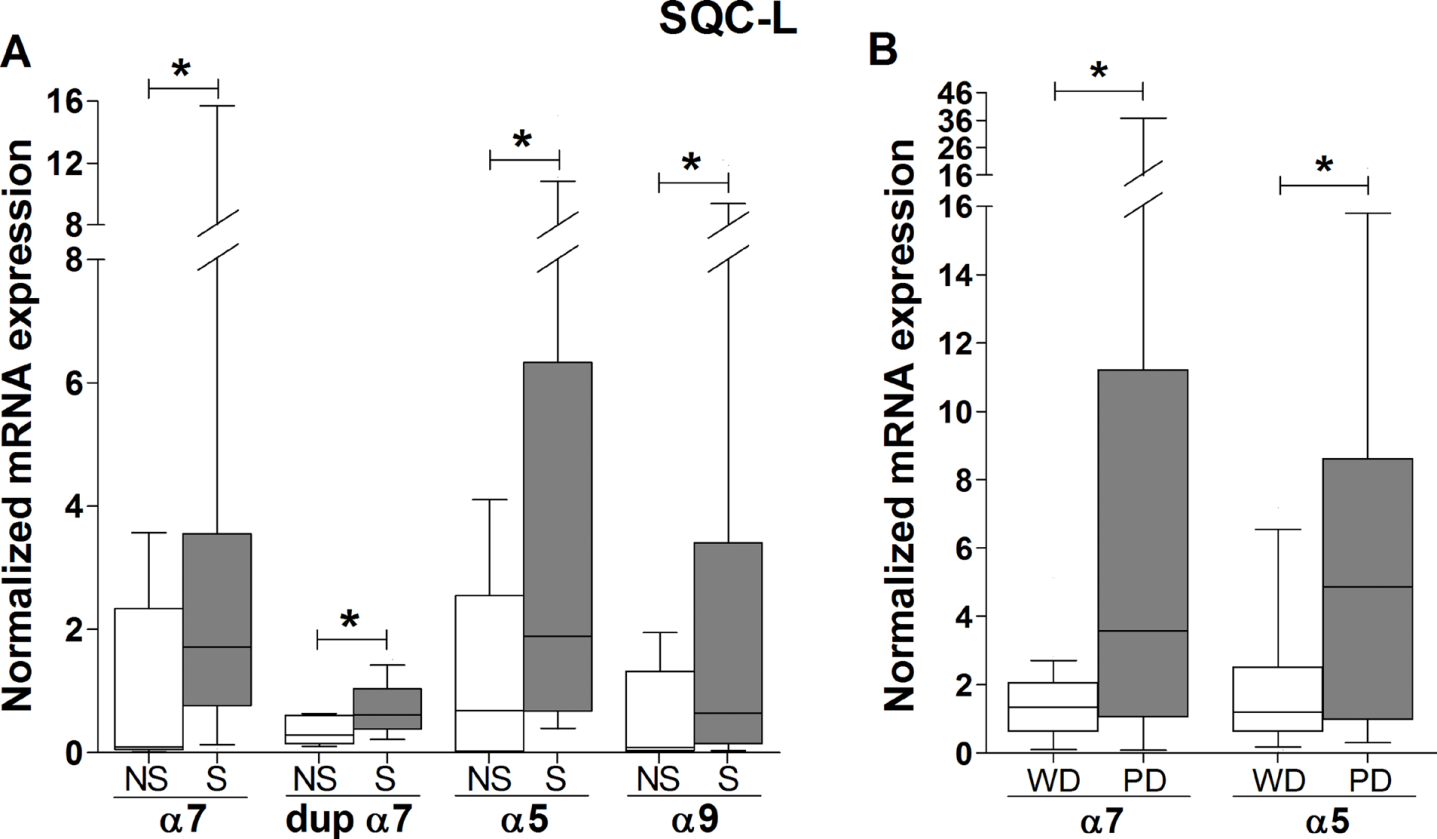
Association of smoking and tumor differentiation grade with the expression levels of nAChR subunit mRNAs in lung tumor biopsies from SQC-L patients Box-plots showing the median and interquartile range of those mRNAs whose tumor expression was significantly affected by smoking (**A**) or tumor grade differentiation (**B**) in SQC-L patients [35 smokers (S) versus 5 non-smokers and second-hand smokers (NS); 22 well- or moderately-differentiated (WD) versus 18 poorly-differentiated (PD) tumors]. The data were analyzed using Student's *t* test with unequal variance assumption (Welch's correction) (panel A) or Mann-Whitney non-parametric test (panel B); *p < 0.05 after comparing the indicated values.

SQC-L is the type of NSCLC most strongly associated with cigarette smoking, while ADC-L, as well as being the most common lung cancer in “never-smokers”, is the one that shows the weakest association. Thus, we investigated whether tobacco exposure modified the expression profile of the nAChR subunit mRNAs in the tumors differently, depending on their histologic type. Figure 3A shows only those mRNAs whose normalized expression in SQC-L tumors appears to have been significantly affected by tobacco. Note the higher expression of all the affected nAChR subunits (α7, dupα7, α5 and α9) in smokers compared to non-smokers, indicating that tobacco components up-regulate the expression of a few nAChR subunit genes in this histologic type. In contrast, none of the nAChR subunit mRNAs tested in patients with ADC-L showed changes in their expression level that could be associated to tobacco use (data not shown).

We also explored whether the degree of tumor differentiation in either histologic type could be associated to changes in the expression patterns of the nAChR subunit mRNAs. Figure 3B shows that only two subunits (α7 and α5) showed significantly increased expression in SQC-L tumors with a poor prognosis. However, the degree of tumor differentiation did not alter the gene expression of any of the nAChR subunits assayed in ADC-L patients (data not shown). No significant gender-related expression differences were found for any of the nAChR subunit genes tested in either of the two tumor types.

### Gene expression pattern of different nAChR subunits in tumors of NSCLC patients and their relationship with survival

**Figure 4:**
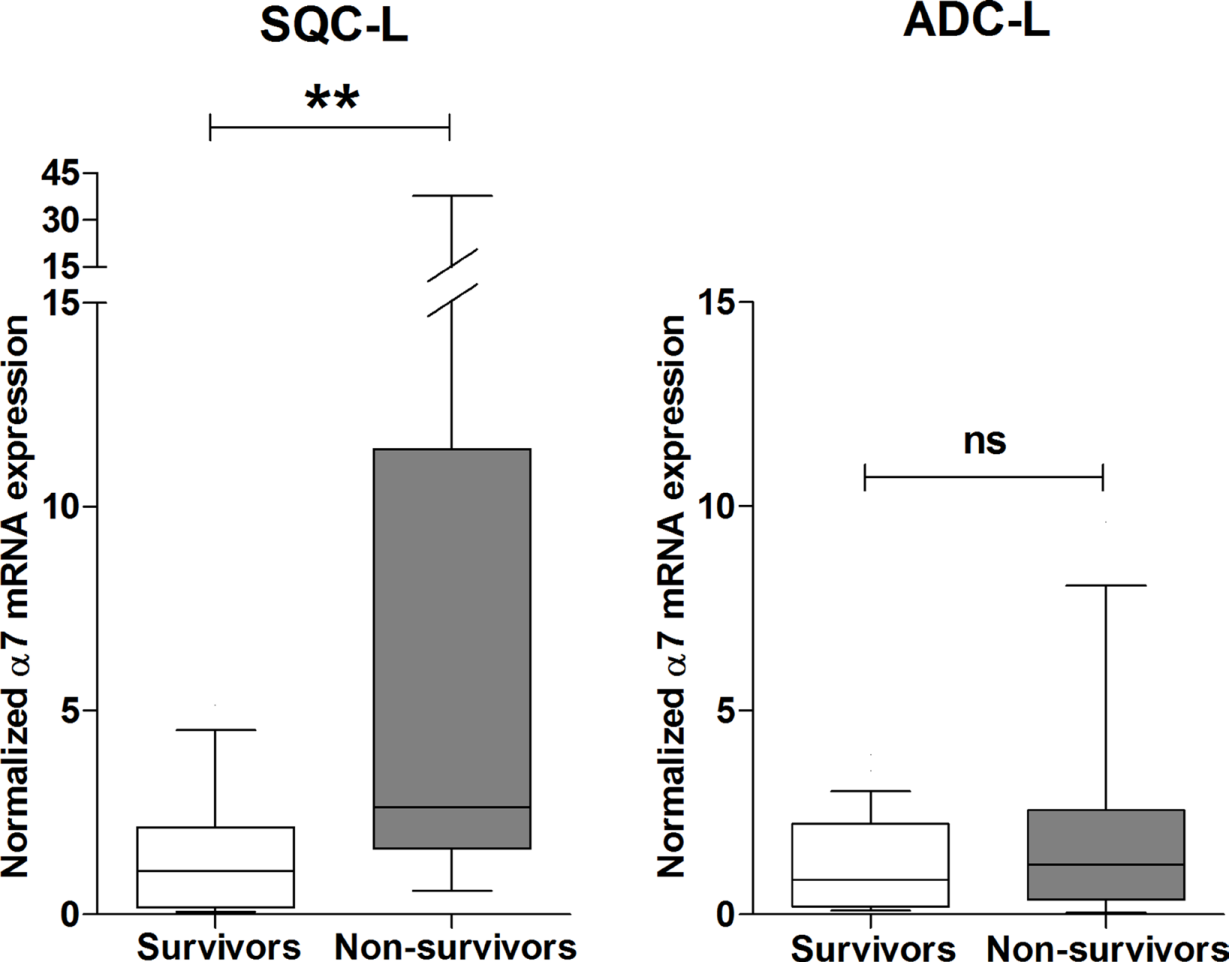
High expression level of α7 mRNA negatively influences survival of patients with SQC-L but not those with ADC-L Box-plots show the median and interquartile range of α7 mRNA levels in tumor biopsies from patients with SQC-L or ADC-L surviving or not at 5-year after surgery. Unlike the rest of the analyzed genes, the only significant difference found was associated with α7 gene expression in SQC-L non-survivors (*n* = 16) and survivors (*n* = 24). In contrast, no significant changes in tumor expression were observed between ADC-L non-survivors (*n* = 12) and survivors (*n* = 26) for any of the nAChR subunit gene. The data were analyzed using the Mann-Whitney non-parametric test; **p < 0.01 after comparing the indicated values; ns = not significant.

Next, we investigated possible differences in the expression pattern of nAChR subunit mRNAs between patients with SQC-L or ADC-L who survived or died within 5 years after surgery. Among all the subunits analyzed, only α7 mRNA levels in SQC-L tumors showed significant differences between survivors and non-survivors. Thus, SQC-L patients who died presented significantly higher tumor α7 mRNA levels than those who survived. In contrast, none of the nAChR subunit mRNAs, including α7, showed any difference in expression level between ADC-L survivors and non-survivors (Figure 4, right).

## DISCUSSION

This study reveals, for the first time, the existence of differences and similarities in the expression pattern of nAChR subunit genes between lung tumor biopsies from patients with SQC-L or ADC-L. Compared with their paired non-tumorous lung specimens, the most substantial differences are the up-regulation of *CHRNA7* expression in SQC-L, particularly in smokers and non-survivors, and the down-regulation of *CHRNA4* in ADC-L. Our study also provides novel data on negative regulation of *CHRFAM7A* expression in SQC-L and ADC-L tumors; this gene encodes the new dupα7 nAChR subunit. Both types of tumors also share a transcriptional dysregulation of the three nAChR subunit genes grouped at the chromosomal locus (15q25.1) that predisposes to lung cancer; gene expression was reduced (*CHRNA3*) or increased (*CHRNA5* and *CHRNB4*). Interestingly, in addition to *CHRNA7*, only a few other nAChR subunit genes modified their expression due to the effect of tobacco (*CHRFAM7A*, *CHRNA5*, *CHRNA9*) or to the degree of tumor differentiation (*CHRNA5*), but these changes only happen in SQC-L tumors. Next, we discuss the most relevant results of the present study.

Lung cancer is the leading global cause of cancer deaths, with NSCLC predominating [28]. Cells from these tumors express several nAChR subtypes whose activation by tobacco components (i.e. nicotine and its carcinogenic derivatives nitrosamines) regulates cell proliferation and apoptotic evasion [see Ref. (12, 18) and references therein]. Based on these observations, it has been proposed that a dysregulation of expression for genes encoding nAChR subunits and/or a dysfunction of these receptors in lung tumors might be involved in cancer-related processes. Several qPCR studies performed in NSCLC cell lines or in primary tumor biopsies from NSCLC or ADC-L patients have revealed that only a few nAChR subunit genes undergo dysregulation during the carcinogenic process [29]. As far as we know, no study in primary tumors from NSCLC patients has yet analyzed whether its two major histologic types (SQC-L and ADC-L) differ in their nAChR subunit gene expression pattern. Our study fills this gap and incorporates two substantial improvements in experimental design compared to previous studies in primary NSCLC tumors. The first is to include sufficient SQC-L and ADC-L patients in the same study as to be able to find, under the same experimental conditions, differences in expression of nAChR subunit genes between the two types of tumor. Secondly, the fact that we have had access to non-tumor lung specimens for all our patients has made it possible to detect fine changes in nAChR subunit gene expression between the tumor and its paired non-tumor sample as well as between both types of tumors.

In a previous study measuring the expression of 11 nAChR subunit genes in 66 NSCLC primary tumors (54 ADC-L/6 SQC-L/5 LCLC, 1 adenosquamous) surgically resected from Chinese patients, only two of the genes evaluated showed significant expression changes in the tumor (increased *CHRNB4* and decreased *CHRNA4*) compared to expression levels in non-tumor tissues [29]. The findings of transcriptional dysregulation of both genes in NSCLC tumors concur with our results showing a significant increase of β4 mRNA levels in tumors from SQC-L and ADC-L patients as well as a dramatic reduction of α4 mRNA levels in ADC-L tumors, the major histologic tumor group included in the Lam et al. study. However, our results differ from those of their study in that we were able to observe a larger number of nAChR subunit genes undergoing expression changes due to the tumorigenic process. Some of these changes are dependent on the histologic tumor type (increased α7 and decreased β2 mRNA levels in SQC-L tumors) while others were shared by SQC-L and ADC-L tumors (increased α5 and β4 and decreased dupα7 and α3 mRNA levels). Moreover, the significant increase of α7 mRNA levels in SQC-L but not in ADC-L tumors found in our study when mRNA expression was determined in a normalized manner (with respect to the paired non-tumor piece), was corroborated by the data on absolute expression of α7 mRNA as determined in both histologic tumor types. On the other hand, changes related to α3 and α5 mRNA levels found in SQC-L and ADC-L tumors in our study concur with a previous paper describing transcriptional dysregulation for *CHRNA3* and *CHRNA5* in lung tumor biopsies paired with their corresponding non-tumor specimens from 21 patients with ADC-L [30].

The inability to detect expression changes for α7 and β2 mRNAs in the tumors of NSCLC patients in the Lam study may be due to the small number of SQC-L patients recruited in that study (only 6 out of 66 NSCLC patients) compared to the 40 SQC-L patients included in our study. In addition, the use of the same patient’s non-tumor lung sample as a calibrator in the qPCR could also have increased the sensitivity of the technique to detect changes in nAChR gene expression in the tumors in our study, unlike the Lam study, which used a pool of seven non-tumor samples unrelated to the patients under study. In fact, our finding of increased α7 mRNA level in SQC-L tumors, especially in smokers, but not in ADC-L tumors, is consistent with previous qualitative data on α7 mRNA expression determined by conventional PCR in tumors from 28 SQC-L and 19 ADC-L patients [31].

Once we identified the nAChR subunit genes whose expression were changed by the carcinogenic process in one or another histological tumor type, the next question was whether such dysregulation could positively or negatively influence tumor growth and development. It is interesting to note that overexpression of α7 in SQC-L and ADC-L cell lines as compared to normal human bronchial epithelial cell lines (NHBE) has been extensively reported [29, 32]. Also, it has been shown that nicotine upregulates the expression of *CHRNA7* in SQC-L and NHBE cell lines, both at the mRNA and protein levels [29, 32, 33]. Moreover, nicotine induces proliferative, survival and angiogenic effects mediated by the activation of α7-nAChRs in both types of cell lines [33–35]. In light of all of the foregoing, our results indicate that α7-nAChR would play a prominent role in the oncogenic process of SQC-L tumors given the significant increase of *CHRNA7* expression in this type of tumor, especially in smokers or in poorly differentiated tumors. Since α7-mRNA levels have been correlated to the smoking history of SQC-L patients [33] and the majority of our SQC-L patients (87.5%) are smokers, the increased α7 mRNA levels in SQC-L tumors found in our study were probably associated with our patients’ smoking habits.

Interestingly, it has been recently revealed that the mechanism underlying nicotine-mediated up-regulation of α7-nAChRs in human SQC-L cell lines occurs at the transcription level as the result of recruitment of Sp1-GATA4 or Sp1-GATA6 complexes on the *CHRNA7* promoter, thereby inducing transcription and increasing α7 mRNA expression in these cells [see Ref. 32 and references therein]. Since α4β2-nAChR seems to be one of the major inhibitors of cancer development [18], the significant down-regulation of *CHRNA4* expression in ADC-L tumors that we have found would result in a reduction in the number of nAChRs containing the α4 subunit, and this may favor carcinogenesis in this histological tumor type.

Our study is the first to detect and quantify the expression of the hybrid gene encoding the new dupα7 nAChR subunit in NSCLC tumors. In both tumor types studied here, *CHRFAM7A* expression was significantly lower than in the paired non-tumor lung specimens. If the dupα7 nAChR subunit expressed in these tumors plays a similar role to that found in *Xenopus* oocytes [26], it is possible that deficient dupα7 subunit expression facilitates the oncogenic process mediated by α7-nAChRs, especially in SQC-L where overexpression of this nAChR subtype appears to be determinant to cancer progression. Further experiments evaluating α7-nAChR-mediated tumorigenic processes and their interference by dupα7 in NSCLC cell lines are necessary to verify the above hypothesis.

Given the association between the *CHRNA3*/*CHRNA5*/*CHRNB4* locus and the risk of lung cancer, attempts have been made to elucidate the pathophysiological role of these three genes encoding α3, α5 and β4 nAChR subunits. Expression of the above genes appears to be vital for cell viability in the most aggressive type of lung cancer, SCLC. Thus, overexpression of the clustered nAChR genes has been reported in a human SCLC cell line, DMS-53 [36], and silencing these genes is known to reduce cell viability [12]. Our data showing overexpression of the genes encoding α5 and β4 subunits in SCQ-L and ADC-L tumors agree with data obtained in SCLC cell lines. However, unlike SCLC cell lines, the *CHRNA3* that encodes the α3 subunit is clearly downregulated in the two NSCLC types analyzed in our study. The opposite results for *CHRNA3* expression in SCLC cell lines and in NSCLC tumors suggests the existence of different regulatory mechanisms for gene expression in these two categories of lung cancer. In fact, like us, other authors have found that expression of the gene encoding the α3 subunit is halved in lung adenocarcinoma as compared with normal lung tissue [30].

Down-regulation of *CHRNA3* expression in the two tumor types in our study could be a consequence of epigenetic silencing of this gene, which is a frequent target of aberrant DNA hypermethylation [37, 38]. Overexpression of *CHRNA3* by ectopic expression induces apoptotic cell death while *CHRNA3* knockdown by short hairpin RNA (shRNA) abolishes the cell response to apoptosis-inducing agents in lung cancer, suggesting this gene is important to human cancer cell survival [37]. Moreover, the interaction of nicotine with nAChRs containing the α5 subunit promotes cell proliferation, angiogenesis and invasion through the activation of the ERK and Akt signaling pathways, upregulation of HIF-1 signaling and elevation of VEGF [39]. Thus, the downregulation of *CHRNA3* expression combined with upregulation of *CHRNA5* expression in our SQC-L and ADC-L tumors might result in under-representation of the α3-containing nAChRs and over-representation of the α5-containing nAChR subtypes on the cell surface, which could lead to a defective cell death response and again, favor the carcinogenic process.

In summary, we have observed that the number of nAChR subunit genes that undergo expression changes in the two major NSCLC histological types is higher than reported in previous studies. This observation is probably the result of the use of paired non-tumor lung samples for all patients included in the study. The separate analysis of gene expression by nAChR subunits in SQC-L and ADC-L tumors, performed for the first time in a single study, also allows us to reliably establish differences and similarities of gene expression in the two histological tumor types. Although dysregulated gene expression of certain nAChR subunits (α5 increased; α3 and dupα7 decreased) could contribute to the oncogenic process in both tumor types, the two main differences are the under-representation of the cancer-inhibiting α4β2-nAChR subtype in ADC-L tumors and the overexpression of the main cancer-promoting α7-nAChR subtype in SQC-L tumors. These differences could be determinant for the growth of one or the other tumor type. Our results showing higher expression levels of α7 mRNA in SQC-L tumors from smokers than from non-smokers, and the observation that α4 mRNA levels in ADC-L tumors are not affected by smoking, appear to support the stronger association of tobacco with the first tumor type than the second. Finally, our data detailing the expression pattern of the different nAChR subunit genes in SQC-L and ADC-L may enhance the understanding of tumorigenic processes in tobacco-related tumors.

## MATERIALS AND METHODS

### Patients and lung samples

Lung specimens from 78 patients diagnosed with NSCLC (40 SQC-L and 38 ADC-L) were obtained from the IdiPAZ Biobank at the Medical University Hospital La Paz, Madrid. Patients underwent lung tumor resection and systematic lymph node dissection between January 2007 and June 2010. The specimens of tumor lung biopsy from each patient, along with their corresponding paired non-tumor paired lung tissue, were respectively obtained from the center of the tumor and from a non-infiltrated area of the lung distant from the tumor, and were confirmed by histopathological evaluation (7th edition of the TNM Classification for Lung Cancer). Optimal cutting temperature (OCT)-embedded tissue specimens were snap-frozen in liquid nitrogen and stored at −80°C until RNA extraction, and then categorized according to clinical information by the Hospital Biobank. After being duly informed, all patients had given written permission for their samples to be used in research. The Institutional Ethics Committee of our hospital approved the study. Histological grading of NSCLC types was based on the revised World Health Organization (WHO) classification of lung tumors [27]. Lepidic-predominant adenocarcinomas were classified as well-differentiated (G1), papillary-predominant and acinar-predominant adenocarcinomas were classified as moderately-differentiated (G2), while soli-predominant and micropapillary-predominant adenocarcinomas were classified as poorly-differentiated (G3). Squamous cell carcinomas with extensive keratinization are well-differentiated, while those with little to no keratinization were classified as poorly-differentiated. The complete follow-up information was recorded; the last date of follow-up for survivors occurred at the end of July 2015. The five-year survival rate was defined as the percentage of patients surviving five years after surgery. Disease-free survival was defined as the median time (months) between surgery and lung cancer recurrence. The interval between follow-up examinations was every 3 months for the first 2 years and every 4 months for the following 3 years.

### Reverse transcription of RNA and quantitative real-time PCR (qPCR)

Prior to RNA extraction, the tumor or non-tumoral nature of all lung specimens included in the study was histopathologically confirmed. Techniques for RNA extraction from tissues or cells, as well as gene expression assay by qPCR from reverse-transcribed RNA using SYBR green-based assays (Bio-Rad, Hercules, CA) and the ABI Prism 7500 Sequence Detector (Applied Biosystems, Foster City, CA), have been described elsewhere [26, 40]. Briefly, total RNA was extracted from lung biopsy specimens (60 to 200 mg) using the Qiagen RNeasy Mini Kit and cDNAs were generated by reverse transcription of total RNA (300 ng) using Taqman Reverse Transcription Reagents (Life Technologies) according to the manufacturer's instructions. The set of primers listed in Table 3 and the following cycling conditions (95°C for 30 s, followed by 40 cycles at 95°C for 5 s and 60°C for 30 s) were used for PCR amplification of nAChR genes. The next pair of primers [5′-TGATCAAGGGAAAGATGACCA-3′, 5′-AACCCTCTTGCAA-TCGAAAA-3′] was employed to amplify the human D esterase gene (*ESD*) which was selected as an endogenous control for the PCR reaction since it is well-established that this gene is one of the most appropriate to study the gene expression profile in non-small cell lung cancer [41, 42]. All determinations were performed in triplicate and paired tumor and non-tumor biopsy specimens were always analyzed in the same analytical run to avoid between-run variations. Reaction conditions were validated separately for each pair of primers, with single peak dissociation curves produced in each reaction run. The relative changes in mRNA expression in tumor biopsies for all nAChR subunits were assessed by the 2^−ΔΔCt^ method, using their corresponding non-tumor paired specimens as calibrator. The number of α7 and dupα7 mRNA copies expressed in paired tumor and non-tumor biopsies were determined using a six-point standard curve prepared with the plasmids pcDNA3.1α7 and pcDNA3.1dupα7-myc-His (our own laboratory preparation) as templates and applying the following formula: number = (ng * number/mole)/(bp * ng/g * g/mole of bp) (cite website: http://cels.uri.edu/gsc/cndna.html).

**Table 3:** List of primers used to amplify the nAChR subunit genes

Subunit (reference gene)	Sequences (5′–3′)	Amplicon size (bp)	Tm (°C)	GC content (%)	Reference
**α3 *(CHRNA3)***	CAGAGTCCAAAGGCTGCAAG	149	65	55	Lips et al. 2005
	AGAGAGGGACAGCACAGCAT		64	55	
**α4 *(CHRNA4)***	GTGGATGAGAAGAACCAGATGATG	74	59	46	Lam et al. 2007
	CAGCGCAGCTTGTAGTCGTG		62	60	
**α5 *(CHRNA5)***	CTTCACACGCTTCCCAAACT	187	64	50	Lips et al. 2005
	CTTCAACAACCTCACGGACA		64	50	
**α7 *(CHRNA7)***	GCTGCAAATGTCTTGGACAGAT	70	59	45	Cedillo et al. 2014
	AACAGTCTTCACCCCTGGATAT		59	45	
**dupα7 *(CHRFAM7A)***	CAATTGCTAATCCAGCATTTGTGG	102	59	42	Present paper
	CCCAGAAGAATTCACCAACACG		60	50	
**α9 *(CHRNA9)***	AAAGATGAACTGGTCCCATTCCT	118	60	43	Lam et al. 2007
	AAGGTCATTAAACAACTTCTGAGCATAT		60	32	
**β2 *(CHRNB2)***	CTGGATCCTTCCCGCTACAAC	146	60	57	Lam et al. 2007
	TGGGTCAGCCAGACATTGGT		61	55	
**β4 *(CHRNB4)***	TCACAGCTCATCTCCATCAAGCT	100	62	48	Lam et al. 2007
	CCTGTTTCAGCCAGACATTGGT		61	50	

### Statistical analysis

Differences in nAChR mRNA levels between tumor and non-tumor paired specimens from NSCLC patients were assessed by the Wilcoxon test. The Mann-Whitney test was used to assess differences between SQC-L or ADC-L tumors in regard to the α7/dupα7 mRNA ratio. The Spearman coefficient was used to analyze the correlation between expression levels of α7 mRNAs and other nAChR subunit mRNAs in tumor biopsies from NSCLC patients. The relationship between two related variables (α7 mRNA expression *vs* expression of each of the remaining nAChR subunits) in the tumor as compared to its paired non-tumor sample was analyzed by Fisher's exact test. A normal distribution was assumed for groups with more than 30 patients (Central Limit Theorem) to compare expression for different nAChR subunit genes between groups of patients with different histological stages, smoking status and clinical outcome within 5 years after surgery. To perform the above comparisons in groups smaller than 30 patients, the Kolmogorov-Smirnov test was applied to evaluate the normality of the data; Student's *t* test with unequal variance assumption (Welch's correction) or Mann-Whitney non-parametric test were applied for normal or non-normal distribution, respectively. A *p* value < 0.05 was considered statistically significant. Analysis was performed with the SPSS software package (version 17.0) and Graphpad Prism 5.0.

## Abbreviations

ADC-L: lung adenocarcinoma
LCLC: large-cell lung carcinoma
nAChR: neuronal nicotinic acetylcholine receptor
NNK: nicotine-derived nitrosamines 4-(methylnitrosamino)-1-(3-pyrydyl)-1-butanone
NNN: N-nitrosonornicotine
NSCLC: non-small cell lung carcinoma
qPCR: real time quantitative polymerase chain reaction
SCLC: small-cell lung carcinoma
SQC-L: squamous cell carcinoma of the lung
